# Impact of Body Mass Index on All-Cause Mortality in Adults: A Systematic Review and Meta-Analysis

**DOI:** 10.3390/jcm13082305

**Published:** 2024-04-16

**Authors:** Marcin M. Nowak, Mariusz Niemczyk, Sławomir Gołębiewski, Leszek Pączek

**Affiliations:** 1 Department of Interventional Cardiology and Internal Diseases, Military Institute of Medicine—National Research Institute, 05-120 Legionowo, Poland; 2 Department of Immunology, Transplant Medicine, and Internal Diseases, Medical University of Warsaw, 02-091 Warszawa, Poland; mariusz.niemczyk@wum.edu.pl (M.N.); leszek.paczek@wum.edu.pl (L.P.)

**Keywords:** all-cause mortality, body-mass index, BMI, cardiovascular disease, obesity

## Abstract

**Background:** Obesity is a risk factor for many diseases, diagnosed by calculating body mass index (BMI). **Methods:** To find an association between BMI and mortality in adults, we searched PubMed for articles published in the 21st century. Our review included 82 original studies, comprising 2.7 million patients and 23.4 million patient years. **Results:** The meta-analysis showed a U-shaped relationship between BMI and all-cause mortality risk, with the lowest mortality in the BMI range of 25–30 kg/m^2^. Subgroup analysis showed a J-shaped relationship, with greater risk in the highest BMI range (>35 kg/m^2^). Among the elderly, BMI values <20 kg/m^2^ were associated with the highest risk. Among diabetic patients, a U-shaped relationship was noticed, again with the highest risk in the lowest (<20 kg/m^2^) and highest BMI range (>35 kg/m^2^). Among patients with cardiovascular disease, the risk increased with BMI values <25 kg/m^2^ but did not noticeably change for BMI exceeding that value. Among cancer patients, the relationship was less pronounced than in other subgroups, with a slightly higher risk (>35 kg/m^2^). **Conclusions:** Our results show that the lowest mortality is observed among patients with BMI 25–30 kg/m^2^. Reduction of body mass should not be a universal recommendation in clinical practice, but it should be individualized.

## 1. Introduction

According to the definition of the World Health Organization (WHO), obesity is a chronic disease characterized by excessive fat deposits that impair health. It is diagnosed by measuring weight and height to calculate body mass index (BMI) [1]. In other words, obesity refers not only to excessive body weight, but also to abnormal body composition. Obesity is a chronic inflammatory disease in which dysfunctional adipocytes secrete inflammatory cytokines and immune cells infiltrate adipose tissue, which results in low-grade inflammation which negatively impacts the whole body [2]. Consequently, obesity is a well-known risk factor for many different diseases, including arterial hypertension (HT) [3], cardiovascular disease (CVD) [4], type 2 diabetes mellitus (T2DM) [5], obstructive sleep apnea (OSA) [6]. and various types of cancer [7]. On the other hand, being underweight is also associated with negative health effects [8]. Since the early 1970s, BMI has been considered an optimal measure of body mass disturbances [9]. In fact, BMI below 18.5 or above 24.9 has been associated with reduced life expectancy [10].

However, there are some clinical conditions in which the paradigm of optimal BMI between 18.5–24.9 fails. For example, in patients with advanced or end-stage chronic kidney disease (CKD), BMI exceeding 24.9 was associated with lower mortality [11]. Similarly, mortality risk of diabetes has been shown to be lower in obese patients compared to normal weight patients [12]. Additionally, obese patients show improved outcomes after immune checkpoint therapy for some types of breast cancer [13]. It was also observed in ever-smokers with chronic obstructive pulmonary disease (COPD) that being overweight or obese is connected to lower hazard of death [14]. This phenomenon is known as the “obesity paradox”, and clearly shows that the association between body mass and mortality is much more complex than previously thought. There are some theories explaining the “obesity paradox”. According to some, it is observed because lean subjects included into studies are more severely ill compared to obese ones. In these cases, low body mass would be attributable to cachexia, which is a complication of a chronic illness [15]. In fact, malnutrition and underweight, similar to obesity, is usually associated with inflammation and deterioration of the function of immune system [16]. Other theories suggest that, in obese patients, adipose tissue bonds lipophilic toxins, preventing their toxicity to the organs of the body [17]. Finally, the “obesity paradox” was proposed to be an effect of methodological flaws [18].

Therefore, we have performed a systematic review of the available literature data with a meta-analysis to search for an association between BMI and mortality in the general population and selected subgroups of patients.

## 2. Materials and Methods

### 2.1. Protocol and Registration

The systematic review protocol was created following the guidelines outlined in the Preferred Reporting Items for Systematic Review and Meta-analysis Protocols (PRISMA) [13]. Additionally, the study protocol was registered prospectively with PROSPERO under the identifier CRD42023421069. There were slights amendments to published protocol.

### 2.2. Data Sources and Searches

In this systematic review and meta-analysis, we conducted a comprehensive search of the PubMed electronic database for articles published in English from May 2000 to May 2023. Our most recent search was carried out on 5 May 2023. We employed a set of relevant keywords, either individually or in combinations, to locate pertinent data. The details of our search strategy, including the number of retrieved articles, can be found in the study protocol in Supplementary Materials. For abstracts that potentially met our inclusion criteria, we obtained the full-text publications. Two independent reviewers evaluated each study for eligibility based on the criteria outlined in the study protocol. We also briefly documented the reasons for any exclusions.

### 2.3. Study Selection

We considered studies eligible if they provided adjusted hazard ratios (HR) for all-cause mortality over a period of at least 12 months in real-world cohorts of individuals with varying BMI. Our inclusion criteria were limited to studies conducted exclusively among adults.

### 2.4. Data Extraction and Quality Assessment

Two independent investigators (MMN and MN) collected the following variables: adjusted hazard ratios (HR), sample sizes, percentages of males, mean ages, average follow-up durations (mean or median), death counts, and adjustment factors. Any discrepancies in their findings were resolved through consensus. The total follow-up duration was acquired from the published materials or computed by multiplying the average follow-up duration by the cohort size (patient years). The number of deaths per 1000 patient years was extracted from the publications or calculated by dividing the number of deaths by the total follow-up duration.

### 2.5. Data Synthesis and Analysis

We used a two-stage, random-effects, dose–response meta-analysis to investigate the relationship between BMI values and hazard ratios for all-cause mortality [19]. The estimates from individual studies were combined using the restricted maximum likelihood method. Because previous evidence suggested a non-linear relationship, we added a quadratic term in all models. Non-linearity was tested with the Wald test. BMI of 25 kg/m^2^ served as reference. For each BMI category, the average of the lower and upper bound was calculated and assigned to a respective hazard ratio. For open-ended BMI categories, we first calculated a mean range for all closed categories in each study, and then we subtracted half of that range from the lowest BMI category bound and added that same value to the highest BMI category bound. The main meta-analysis included all studies. Subgroup meta-analyses were carried out for the general population, the elderly, patients with diabetes, patients with cardiovascular diseases, and patients with cancer. Also, we performed subgroup analysis for Asian and non-Asian populations. The I2 statistic and the Cochran Q-test were used to assess heterogeneity. The analyses were carried out in the R software (v. 4.2.1), with the “dosresmeta” package for all meta-analyses [20]. Two independent authors conducted a risk of bias assessment for the included studies utilizing the Newcastle-Ottawa Scale (NOS). The NOS encompasses three domains: (1) selection, (2) comparability, and (3) outcome.

## 3. Results

**Figure 1:**
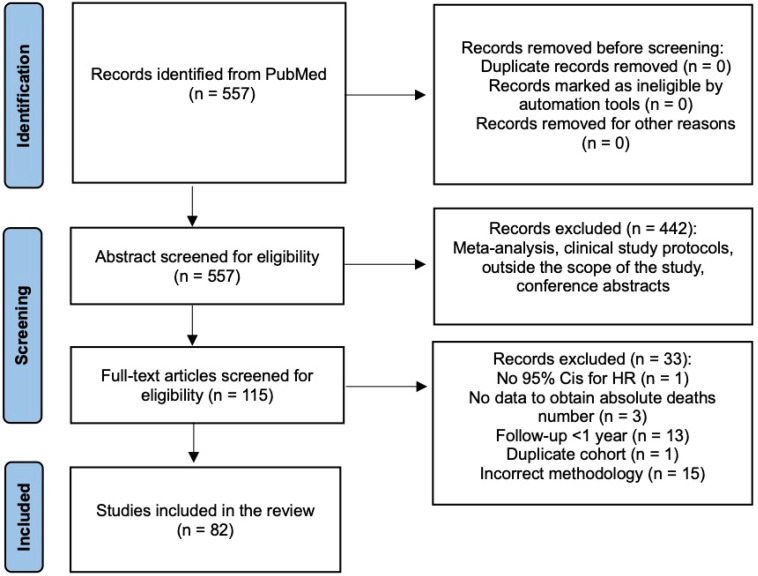
Preferred Reporting Items for Systematic Reviews and Meta-Analyses (PRISMA) flow chart of the study selection process.

A total 557 publications were identified, and 115 that were potentially eligible were retrieved in full text. Finally, 82 studies (97 cohorts) were included in the review (Figure 1). Risk of bias assessment using NOS showed that the studies’ quality was moderate. In the analysis, we included two cohorts from eight studies [21,22,23,24,25,26,27,28] and three cohorts from one study [29]. The characteristics of all included studies are summarized in Supplementary Table S1.

**Figure 2:**
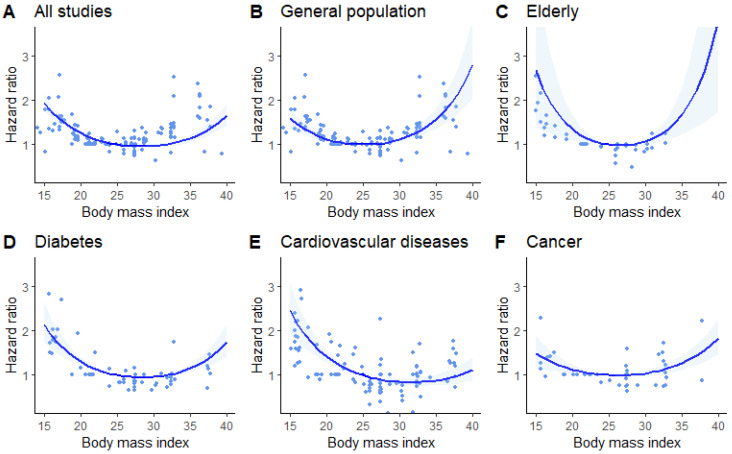
Dose–response meta-analysis of studies investigating the association between body mass index (BMI) and risk of all-cause mortality. BMI of 25 kg/m^2^ served as reference. Ribbons show 95% confidence intervals. Dots are estimates from individual studies. (**A**)—Univariate Cochran Q-test for residual heterogeneity: *p*-value < 0.001, I-square statistic = 91.7%; (**B**)—Univariate Cochran Q-test for residual heterogeneity: *p*-value < 0.001, I-square statistic = 86.1%; (**C**)—Univariate Cochran Q-test for residual heterogeneity: *p*-value < 0.001, I-square statistic = 81.7%; (**D**)—Univariate Cochran Q-test for residual heterogeneity: *p*-value < 0.001, I-square statistic = 84.3%; (**E**)—Univariate Cochran Q-test for residual heterogeneity: *p*-value < 0.001, I-square statistic = 91.5%; (**F**)—Univariate Cochran Q-test for residual heterogeneity: *p*-value < 0.001, I-square statistic = 73.9%.

The dose–response meta-analysis of all 97 cohorts showed a U-shaped relationship between BMI and all-cause mortality risk (*p* < 0.001 for non-linearity), with the lowest mortality in the BMI range of 25–30 kg/m^2^ (Figure 2A). Heterogeneity was substantial (I2 = 91.7%, Cochran Q-test *p* < 0.001). In the general population, the relationship between BMI and risk of all-cause mortality in the general population was J-shaped (*p* < 0.001 for non-linearity), with greater risk for all-cause mortality in the highest BMI range (>35 kg/m^2^, Figure 2B, Table 1). Heterogeneity remained substantial (I2 = 86.1%, *p* < 0.001).

Among the elderly, BMI values below 20 kg/m^2^ were associated with the highest risk for all-cause mortality (*p* < 0.001 for non-linearity) (Table 1), with no studies reporting data for BMI above 35 kg/m^2^ (Figure 2C, I2 = 81.7%, *p* < 0.001).

Among patients with diabetes, the risk for all-cause mortality was the greatest in the lowest BMI range (<20 kg/m^2^), but was also increased, albeit to a lesser extent, in the BMI range above 35 kg/m^2^ (Table 1) (*p* < 0.001 for non-linearity, Figure 2D, I2 = 84.3%, *p* < 0.001).

Among patients with cardiovascular diseases, the risk for all-cause mortality increased with BMI values below 25 kg/m^2^ but did not change noticeably for BMI values above that value (Table 1) (*p* < 0.001 for non-linearity, Figure 2E, I2 = 91.5%, *p* < 0.001).

Among patients with cancer, the relationship between BMI and all-cause mortality was less pronounced than in other subgroups, with a slightly higher risk for all-cause mortality within the extreme BMI values (Table 1) (*p* < 0.001 for non-linearity, Figure 2F, I2 = 73.9%, *p* < 0.001).

**Figure 3:**
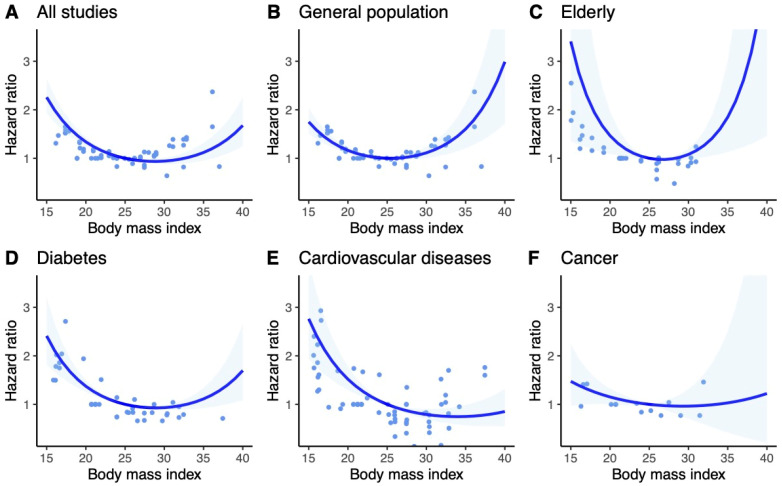
Dose–response meta-analysis of studies investigating the association between BMI and risk of all-cause mortality in Asian population. BMI of 25 kg/m^2^ served as reference. Ribbons show 95% confidence intervals. Dots are estimates from individual studies. (**A**)—Univariate Cochran Q-test for residual heterogeneity: *p*-value < 0.001, I-square statistic = 87.9% (**B**)—Univariate Cochran Q-test for residual heterogeneity: *p*-value < 0.001, I-square statistic = 82.5% (**C**)—Univariate Cochran Q-test for residual heterogeneity: *p*-value < 0.001, I-square statistic = 84.2% (**D**)—Univariate Cochran Q-test for residual heterogeneity: *p*-value < 0.001, I-square statistic = 72.4% (**E**)—Univariate Cochran Q-test for residual heterogeneity: *p*-value < 0.001, I-square statistic = 91.0% (**F**)—Univariate Cochran Q-test for residual heterogeneity: *p*-value < 0.001, I-square statistic = 85.6%.

**Figure 4:**
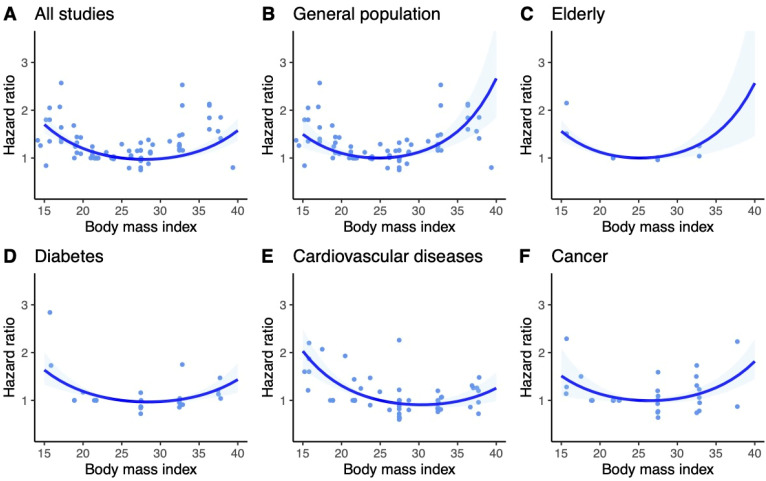
Dose–response meta-analysis of studies investigating the association between BMI and risk of all-cause mortality in non-Asian population. BMI of 25 kg/m^2^ served as reference. Ribbons show 95% confidence intervals. Dots are estimates from individual studies. (**A**)—Univariate Cochran Q-test for residual heterogeneity: *p*-value < 0.001, I-square statistic = 87.0% (**B**)—Univariate Cochran Q-test for residual heterogeneity: *p*-value < 0.001, I-square statistic = 86.8% (**C**)—Univariate Cochran Q-test for residual heterogeneity: *p*-value < 0.001, I-square statistic = 0.0% (**D**)—Univariate Cochran Q-test for residual heterogeneity: *p*-value < 0.001, I-square statistic = 60.9% (**E**)—Univariate Cochran Q-test for residual heterogeneity: *p*-value < 0.001, I-square statistic = 91.5% (**F**)—Univariate Cochran Q-test for residual heterogeneity: *p*-value < 0.001, I-square statistic = 69.0%.

The results for Asian and non-Asian populations were consistent with the general findings. However, in the subgroup of the elderly within the Asian population, a more pronounced U-shaped trend was observed compared to the general and non-Asian populations. Subgroup analyses of Asian and non-Asian populations are included in Figure 3 and Figure 4.

## 4. Discussion

We evaluated evidence from real-world studies performed during the last two decades that assessed association between BMI and all-cause mortality. To improve the precision of results, our analysis was limited to studies in which HR was presented. The meta-analysis of all included studies showed a U-shaped relationship between BMI and all-cause mortality risk, with the lowest mortality in the BMI range of 25–30 kg/m^2^.

We observed higher risk of all-cause mortality in the highest BMI range in the general population. Among the elderly, BMI values below 20 kg/m^2^ were associated with the highest risk. No studies reported data for BMI above 35 kg/m^2^. We found the highest risk in not only the lowest (<20 kg/m^2^), but also in the highest BMI range (>35 kg/m^2^) in diabetic patients. In cardiovascular disease patients, the risk increased with BMI values below 25 kg/m^2^, but did not noticeably change for BMI values above that value. Among cancer patients, the relationship was less pronounced than in other subgroups, with a slightly higher risk exceeding the extreme BMI values. Of note, substantial heterogeneity was noted both in the main analysis and in subgroup analyses. Therefore, the overall evidence on the association of BMI with all-cause mortality in real-world clinical settings is of suboptimal quality.

Our findings are inconsistent with previous opinions, which state that the optimal BMI was between 18.5–24.9 kg/m^2^ [10]; however, in a large systematic review with meta-analysis, including 30 million participants, the lowest mortality was observed in people with a BMI of 25 [30]. Our analysis was intentionally limited to papers published in the 21st century. Due to the growing awareness of healthy lifestyle principles and the advances of medicine, demographic changes have taken place worldwide. As a result, the aging of societies, along with changes in age distribution of populations, are observed [31]. This is followed by changes in epidemiological significance of diseases. For example, chronic kidney disease, in which the “obesity paradox” has been reported, is observed in almost 15% of US population [32]. Additionally, renal diseases were the 10th most common reason of death in 2019 worldwide [33], but they are expected to reach the 5th position by 2040 [34]. These changes may impact the relationship between body mass and mortality. One possible explanation of differences between our results and earlier studies is that a large proportion of included studies were conducted on special populations, which were further assessed in subgroup meta-analyses. However, we must realize that these special populations become increasingly frequent. People with cardiovascular disease, diabetes, or chronic kidney disease have become a substantial part of society, and it should be noted that undue reduction in their body weight may be harmful. Similarly, the proportion of older people and patients with cancer is also increasing. Frailty, which is often marked by a reduction in muscle mass and body weight, is known to be a risk factor of unfavorable outcome [35].

Another possible explanation of our results is that the body composition, not the body mass index itself, is the main determinant of mortality. Body composition analysis provides a more comprehensive assessment by analyzing the specific proportions of fat, muscle, bone, and water in the body. High body fat percentage, particularly visceral fat, has been linked to increased mortality risk due to its association with various health conditions such as cardiovascular disease, diabetes, and certain cancers. Conversely, a higher proportion of lean muscle mass might be associated with better health outcomes.

Research suggests that body composition measures, particularly high levels of body fat and low levels of muscle mass, may offer more nuanced insights into mortality risk compared to BMI alone. While both BMI and body composition play roles in assessing health risks, body composition analysis might provide a more detailed understanding of an individual’s health profile concerning mortality risk due to its consideration of fat distribution and muscle mass, among other factors.

As mentioned above, low muscle mass, being a part of frailty, is associated with increased mortality, and an increase in muscle mass will possibly improve the outcome. Then, an athletic body with BMI exceeding 25 may be connected to better outcome compared with people with BMI within “normal” range. This thesis is consistent with a finding that survival rates are increased in aerobic athletes compared to the general population [36], and may explain, at least in part, our results.

Limitations of our meta-analysis should also be acknowledged. (1) The quality of available data is far from optimal. (2) Only selected subpopulations were analyzed in our meta-analysis, and data on optimal BMI in many other clinical conditions remain unclear. (3) Our analysis omits changes of BMI during a patient’s life, which may also be important. For example, in so-called reverse causality, the decrease in body mass may be caused by underlying illness, and it is, therefore, not connected to improved survival [37,38]. (4) Differences in the follow-up (from 1.2 to 20 years) and the mean age of the initiation of the follow-up may also influence the results. (5) Differences in adjusted factors (Supplementary Table S1). (6) Differences in BMI normal values: the standard BMI range for individuals in Asia is 20–23, with 23–25 indicating overweight and >25 kg/m^2^ indicating obesity. However, the difference between the general BMI categories and those specific to Asians amplifies the impact of our study findings. Additionally, diversity of effects achieved in different subpopulations limits the clinical utility of our results; in clinical settings, different clinical conditions frequently coexist. Thus, further investigations are needed to facilitate clinical decision-making in the future. (7) Another limitation of our study is that we focused only on BMI, whilst it is known that BMI fails to fully capture cardiometabolic risk. This is, at least partially, because BMI is an insufficient marker of abdominal adiposity. On the other hand, abdominal adiposity may be simply assessed with the measurement of waist circumference, which correlates with mortality. However, the association of waist circumference with morbidity and mortality becomes evident after adjustment for BMI. Therefore, both metrics provide complementary information, and should be used together to achieve the most predictive information [39]. An additional metric that may be useful in predicting cardiometabolic risk is waist-to-height ratio, which was also omitted in our analysis [40].

Our results show that with current demographic changes, the lowest mortality is observed among patients in the BMI range of 25–30 kg/m^2^. However, target body mass should be assigned individually based on clinical conditions in particular patients. Reduction of body mass should not be a universal recommendation in clinical practice, but it should instead be individualized depending on patient’s diagnosis; in many clinical situations, it is better to remain slightly overweight. Further studies are required to determine optimal BMI values in particular clinical conditions.

## Figures and Tables

**Table 1:** Predicted hazard ratios for all-cause mortality.

Subgroup	BMI	HR	95% CI	I2	Q-Test	Wald Test
General population	15	1.60	1.39–1.84	86.1%	*p* < 0.001	*p* < 0.001
	20	1.13	1.07–1.18			
	25	1.00	1.00–1.00			
	30	1.12	1.06–1.18			
	35	1.58	1.35–1.84			
	40	2.80	2.05–3.82			
Elderly	15	2.68	1.40–5.11	81.7%	*p* < 0.001	*p* < 0.001
	20	1.36	1.10–1.68			
	25	1.00	1.00–1.00			
	30	1.07	1.00–1.15			
	35	1.68	1.23–2.29			
	40	3.84	1.74–8.44			
Diabetes	15	2.14	1.73–2.65	84.3%	*p* < 0.001	*p* < 0.001
	20	1.31	1.22–1.40			
	25	1.00	1.00–1.00			
	30	0.96	0.94–0.98			
	35	1.15	1.07–1.24			
	40	1.74	1.41–2.14			
CVD	15	2.45	1.95–3.09	91.5%	*p* < 0.001	*p* < 0.001
	20	1.42	1.29–1.57			
	25	1.00	1.00–1.00			
	30	0.85	0.79–0.92			
	35	0.89	0.77–1.02			
	40	1.11	0.88–1.40			
Cancer	15	1.49	1.17–1.90	73.9%	*p* < 0.001	*p* < 0.001
	20	1.13	1.02–1.24			
	25	1.00	1.00–1.00			
	30	1.04	0.98–1.11			
	35	1.27	1.12–1.44			
	40	1.83	1.46–2.28			
Other	15	2.00	1.55–2.58	82.3%	*p* < 0.001	*p* < 0.001
	20	1.31	1.19–1.45			
	25	1.00	1.00–1.00			
	30	0.88	0.83–0.94			
	35	0.90	0.81–1.01			
	40	1.07	0.89–1.29			

BMI—body-mass index, CI—confidence interval, CVD—cardiovascular disease, HR—hazard ratio.

## Data Availability

Not applicable.

## Supplementary Materials

The following supporting information can be downloaded at: https://www.mdpi.com/article/10.3390/jcm13082305/s1, Table S1 and study protocol.
