# Supplementary material for: Impact of Body Mass Index on All-Cause Mortality in Adults: A Systematic Review and Meta-Analysis
Source: J Clin Med. 2024 Apr 16;13(8):2305. doi: 10.3390/jcm13082305 (PMC11051237; doi:10.3390/jcm13082305)
Supplement: Supplementary file 1 [file jcm-13-02305-s001.zip › jcm-2908400-supplementary.pdf]

no	disease_cat	study	cases	n	disease	continent	follow_up_years	adjustments	Quality of study (NOS)
1	General population	Baldinger et al. 2006	588	22927	adult men with high insurance	Europe	8.2	age, calendar year of observation	good
2	General population	Cohen et al. 2014	11386	239526	African American adults	North America	11.7	sex, education, marital status, alcohol consumption, and physical activity	good
3	General population	Freedman et al. 2006 a	2278	64493	adults	North America	14.7	smoking duration, alcohol consumption, race and education	good
4	General population	Freedman et al. 2006 b	1495	19011	adults	North America	14.7	smoking duration, alcohol consumption, race and education	good
5	General population	Gelber et al. 2007	5438	99253	adults	North America	5.7	baseline age (continuous, in years), alcohol consumption, physical activity	good
6	General population	Hjartåker et al. 2005 a	652	98396	adult premenopausal women	Europe	9.1	age, BMI	good
7	General population	Hjartåker et al. 2005 b	535	4050	adult postmenopausal women	Europe	9.1	age, BMI	good
8	General population	Hu et al. 2022	683	12089	aduts	Asia	5.6	age, sex, current smokers and drinkers, SBP and DBP.	fair
9	General population	Katzmarzyk et al. 2012 a	707	5210	adults	North America	13.9	age, sex, exam year, smoking status, alcohol consumption and education	good
10	General population	Katzmarzyk et al. 2012 b	442	5312	adults	North America	13.9	age, sex, exam year, smoking status, alcohol consumption and education	good
11	General population	Kee et al. 2017	1035	32839	adults	Asia	5	age group, gender, ethnicity	good
12	General population	Klenk et al. 2009 a	8189	85000	adults	Europe	15.1	age, smoking	good
13	General population	Klenk et al. 2009 b	7368	99698	adults	Europe	15.1	age, smoking	good
14	General population	Lin et al. 2011 a	1549	65718	adults	Asia	10	age, smoking status, alcohol intake, betel-nut chewing, level of physical activity, income level and education level	good
15	General population	Lin et al. 2011 b	2398	58738	adults	Asia	10	age, smoking status, alcohol intake, betel-nut chewing, level of physical activity, income level and education level	good
16	General population	Manne-Goehler et al. 2021	779	9728	rural population	Africa	7	adjusted for current age (as timescale), sex, HIV status and socioeconomic status.	g o o d

17	General population	Nagai et al. 2010 a	3685	21038	adults	Asia	12	age in 5-year categories; weight change since age 20 years; education; marital status; cigarette smoking; alcohol drinking; time spent walking per day; sports and physical exercise time per week, history of kidney disease; history of liver disease; history of hypertension and history of diabetes	good
18	General population	Nagai et al. 2010 b	2022	22934	adults	Asia	12	age in 5-year categories; weight change since age 20 years; education; marital status; cigarette smoking; alcohol drinking; time spent walking per day; sports and physical exercise time per week, history of kidney disease; history of liver disease; history of hypertension and history of diabetes	good
19	General population	Shimazu et al. 2009 a	2617	38080	adults	Asia	7	age, sex, smoking status, alcohol drinking, walking duration, education; green vegetables, total fruit and total energy intake, green tea intake	good
20	General population	Shimazu et al. 2009 b	2617	38080	adults	Asia	7	age, sex, smoking status, alcohol drinking, walking duration, education; green vegetables, total fruit and total energy intake, green tea intake	good
21	General population	Song et al. 2007	11081	338320	adult women	Asia	10	Model 2: age, alcohol consumption, smoking, engagement in regular physical exercis, area of residence, height (cm), menopausal status, employment status	good
22	General population	Thorogood et al. 2003	1134	10858	meat eaters and non-meat eaters	Europe	18	age at recruitment, sex, smoking habits, reported pre-existing cardiovascular disease or diabetes	good
23	Diabetes	Zahir et al. 2019 a	73	150	diabetes	Australia	14.6	sex, educational attainment, weekly income, smoking status, physical activity, cluster, and strata of age group and marital status	good
24	General population	Zahir et al. 2019 b	1438	10394	general	Australia	14.6	sex, educational attainment, weekly income, smoking status, physical activity, cluster, and strata of age group and marital status	good
25	Elderly	Atlantis et al. 2010	409	1000	elderly	Australia	12	age, sex, smoking status, instrumental activities of daily living, timed "get-up and go", social activity, cognitive impairment, cardiovascular disease	good
26	Elderly	Corrada et al. 2006	11203	13451	elderly	North America	9.8	age at entry, gender, and smoking	good
27	Elderly	Li et al. 2017	327	885	older men with impaired glucose regulation	Asia	10	age, smoking status, history of hypertension, systolic blood pressure, triglycerides, low-density lipoprotein cholesterol, fasting plasma glucose, 2h-PG, serum creatinine	good
28	Elderly	Takata et al. 2007	49	675	80-year-old population	Asia	4	sex, current outpatient status, smoking, drinking, weight loss, systolic blood pressure, physical activity, functional status, marital status, preexisting diseases, place of residence, and levels of total serum cholesterol and glucose	good
29	Elderly	Tamakoshi et al. 2010 a	588	11230	older adults	Asia	11.2	smoking, drinking, physical activity, sleep duration, stress, education, marital status, green vegetables, stroke, MI, cancer (includes unknown groups)	good
30	Elderly	Tamakoshi et al. 2010 b	3964	15517	older adults	Asia	11.7	smoking, drinking, physical activity, sleep duration, stress, education, marital status, green vegetables, stroke, MI, cancer (includes unknown groups)	good
31	Elderly	Wang et al. 2017	926	2090	elderly	Asia	20	age, sex, smoking and drinking status, pre-existing disease like hypertension, coronary heart disease, rheumatic heart disease and COPD	good
32	Elderly	Wang et al. 2018	5962	8026	elderly over 80 years old	Asia	3.6	age, gender, socio-demographic and lifestyle factors (smoking, alcohol drinking, physical activity, intake of fruit, vegetable, meat, fish and tea), residence (urban/rural) and education	good

33	Diabetes	Costanzo et al. 2015	3744	10568	type 2 diabetes	Europe	10.6	age, sex, duration of diabetes, smoking history, systolic blood pressure, COPD, cancer, CKD, and previous CVD	good
34	Diabetes	Edqvist et al. 2018	17546	149345	type 2 diabetes	Europe	5.5	age group, year of inclusion, sex, the interaction between duration of diabetes and age, income, education, marital status, immigrant status, coronary heart disease, acute myocardial infarction, stroke, atrial fibrillation, heart failure and chronic kidney disease	good
35	Diabetes	German et al. 2020	716	10251	type 2 diabetes	North America	3.7	adjusted for age, gender, race (binary), arm of the trial, statin use, GFR, mean SBP, mean DBP, mean LDL, mean HD, mean HBA1C, years of diabetes, cigarette smoking status, antihypertensive medication use, baseline cardiovascular disease status.	good
36	Diabetes	Hong et al. 2022	4215	53988	type 2 diabetes	Asia	8.6	Adjusted for sex (male, female), age (35–44, 45–54, 55–64, 65–74, 75+), income level (Medical Aid, deciles 1–2, deciles 3–4, deciles 5–6, deciles 7–8, deciles 9–10), residence (Seoul, metropolitan, city and county), smoking status (never smoker, former smoker, current smoker), alcohol intake (never, 2–3 times/month, 1–2 times/week, 3–4 times/week, every day), physical activity (no activity, 1–2 times/week, 3–4 times/week, 5–6 times/week, every day), family history of diabetes mellitus (yes, no), medication adherence (adherence, non-adherence), systolic blood pressure (continuous variable) and total cholesterol (continuous variable).	good
37	Diabetes	Huang et al. 2021	1162	6354	type 2 diabetes	Asia	8	NA	
38	Diabetes	Lee et al. 2018	7360	48438	type 2 diabetes	Asia	10.7	sex, age, systolic blood pressure, fasting glucose, total cholesterol, drinking status, smoking status, physical exercise, household income, use of anti-hypertensive drugs, use of statin drugs, use of anti-platelet drugs, and use of glucose-lowering drugs (metformin, sulfonylurea, insulin, and other glucose-lowering drugs)	good
39	Diabetes	Lee et al. 2021	2847	44055	type 2 diabetes	Asia	3.8	adjusted for age, sex, smoking history, alcohol consumption, regular exercise, and monthly income, s fasting plasma glucose, diabetes duration (≥5 years versus <5 years), and diabetes type (type 1 versus type 2 diabetes).	good
40	Diabetes	Lin et al. 2019	685	8545	type 2 diabetes	Asia	4.03	age, gender, smoking, alcohol drinking, duration of diabetes, type of hypoglycaemic drug, hypertension drug treatment, hyperlipidaemia, DKA, HHNK, severe hypoglycaemia, stroke, myocardial infarction, peripheral neuropathy, intermittent claudication, neuropathy, nephropathy, triglyceride, high-density lipoprotein, low-density lipoprotein, fasting plasma glucose, HbA1C and hospitalization status one year prior to baseline	good
41	Diabetes	Liu et al. 2017	1254	11449	type 2 diabetes	Asia	6.94	age, sex, fasting blood glucose, smoking status, systolic blood pressure, history of hypertension, history of stroke, history of cancer, history of MI	good

42	Diabetes	Ma et al. 2019	996	17259	type 2 diabetes	Asia	7.33	sex, age; duration of diabetes; smoking; micro- and macrovascular complications from diabetes, blood pressure, and albuminuria; LDL-C, HDL-C, triglycerides, HbA1c levels; treatment for hypertension or diabetes; and the use of antiplatelet drrugs or statins	good
43	Diabetes	Orsi et al. 2023	3602	15656	type 2 diabetes	Europe	7.4	for age and sex (Model 1), and age, smoking status, PA level, and comorbidities	good
44	Diabetes	Salehidoost et al. 2018	710	2383	type 2 diabetes	Asia	7.8	age, sex, systolic blood pressure, diastolic blood pressure, fasting plasma glucose, total cholesterol, triglyceride, smoking and diabetes duration	good
45	Diabetes	Vestberg et al. 2018	1261	17449	type 1 diabetes	Europe	8.5	sex, age, diabetes duration, HbA1c, blood pressure (both diastolic and systolic), smoking, microalbuminuria and macroalbuminuria, lipid-lowering agents, blood pressure medications and level of education	good
46	CVD	Aiumtrakul et al. 2022	253	5887	high CV risk subjects	Asia	5	NA	fair
47	CVD	Cai et al. 2014	33	247	advanced heart failure with cardiac resynchronization therapy	Asia	2.5	age, gender, ischemic cardiomyopathy, diabetes mellitus, hypertension, hypercholesterolemia, pulmonary arterial hypertension (PAH), left bundle branch block (LBBB), LVEF, MR severity, serum blood urea nitrogen and creatinine, and baseline use of β-blocker, angiotensin-converting enzyme inhibitor and angiotensin receptor blocker (ACEI/ARB) and statin	good
48	CVD	Curtis et al. 2005	2597	7767	heart failure	North America	3.08	age; sex; LVEF; NYHA class; history of myocardial infarction; dyspnea; duration of HF symptoms; diabetes; hypertension; HF etiology; blood pressure; heart rate; rales; elevated jugular venous pressure; peripheral edema; presence of an S3 heart sound; radiologic evidence of pulmonary congestion; prior use of digoxin, potassium-sparing diuretics, all other diuretics, angiotensin-converting enzyme inhibitors, nitrates, and/or hydralazine; randomization to digoxin; and creatinine levels	good
49	CVD	Faggioni et al. 2018	440	9420	PCI with drug-eluting stents	multinational	3	age, diabetes, hypertension, smoking habit, hypercholesterolemia, family history of CAD, presentation (STEMI, NSTEMI, unstable angina, stable CAD), number of stents implanted, stent length, and B2/C lesions	good
50	CVD	Feng et al. 2021	1195	8551	coronary artery disease	Asia	7.5	age, sex, hypertension, history of MI, acute MI, DM, current smoking, treatment strategy (MT, PCI, or CABG) and preoperative SYNTAX score	good
51	CVD	Haass et al. 2011	874	4109	heart failure	North America, Australia, Asia	4.1	age, sex, New York Heart Association (NYHA) class, heart rate, systolic blood pressure, left ventricular hypertrophy, LVEF, cause of HF, hospitalization for HF within the last 6 months, history of hypertension, myocardial infarction, stroke, chronic obstructive pulmonary disease and/or diabetes, use of diuretics, digoxin, a calcium-channel blocker, lipid-lowering agents, an angiotensin-converting enzyme (ACE) inhibitor, or a -blocker, and N-terminal pro-brain natriuretic peptide	good

52	CVD	Hamaguchi et al. 2010	431	2154	heart failure	Asia	2.1	age, sex, cause of heart failure, medical history (diabetes mellitus, hyperuricemia, smoking), serum creatinine, hemoglobin concentration, NYHA functional class at discharge, BNP at discharge, and medication use	good
53	CVD	Hwang et al. 2022	1732	4146	heart failure	Asia	1.8	age, sex, systolic blood pressure, presence of DM, hemoglobin and GFR levels, and natriuretic peptide level	good
54	CVD	Inoue et al. 2016	159	6379	non-valvular atrial fibrillation	Asia	2	BMI, age, sex, heart failure, hypertension, diabetes mellitus, stroke, coronary artery disease, antiplatelets, cardiomyopathy, permanent atrial fibrialltion, statins, digitalis, Na-channel blockers	good
55	CVD	Jong et al. 2019	74	1221	diabetes mellitus and acute coronary syndrome	Asia	1.52	BMI, age, sex, CKD, left ventricular ejection fraction <40%, left main disease, ACS subtype, percutaneous coronary intervention treatment, coronary artery bypass grafting treatment, hemoglobin A1c, discharge medications (including angiotensin-converting enzyme inhibitors/angiotensin II receptor blockers, beta blockers, dual-antiplatelet medications, or statins), and current cigarette smoking status	good
56	CVD	Kenchaiah et al. 2007	1831	7599	heart failure	multinational	3.14	age, sex	good
57	CVD	Masoudkabir et al. 2022	1838	17751	midterm surgical revascularization	Asia	5	age, sex, diabetes mellitus, hypertension, hyperlipidemia, family history, current smoking, opium abuse, CKD, ejection fractions, left main involvements, numbers of diseased vessels, numbers of grafts, intensive care unit stay, off-pump surgery, myocardial infarction of less than 7 days, chronic obstructive pulmonary disease, cerebrovascular disease, previous percutaneous coronary interventions, discharge medications	good
58	CVD	Moholdt et al. 2017	2372	2821	coronary heart disease	Europe	12.5	age, examination year, smoking status, diabetes mellitus, alcohol consumption, hypertension, health status, physical activity for body mass index and vice versa, and stratified by sex	good
59	CVD	Nezu et al. 2023	437	6927	atrial fibrillation	Asia	3.9	age, sex, smoking status, histories of hypertension, DM, heart failure, coronary heart diseases, chronic hepatic dysfunction, stroke, major bleeding, and malignancy, anemia (hemoglobin<?11 g/dl), renal dysfunction (estimated glomerular filtration rate <30 ml/min/1.73 m2 or hemodialysis), antiplatelets’ use, and statin use	good
60	CVD	Olsen et al. 2008 a	2555	10449	stroke	Europe	1.5	sex	good
61	CVD	Olsen et al. 2008 b	2286	11435	stroke	Europe	1.5	sex	good
62	CVD	Shiga et al. 2018	450	6216	myocardial infarction	Asia	3.6	age, sex, risk factors, left ventricular ejection fraction, and medications	good
63	CVD	Tokarek et al. 2019	24	147	transcatheter aortic valve implantation	Europe	1.26	age, sex	good
64	CVD	Vaduganathan et al. 2012	338	2640	valvular heart surgery	North America	2.98	age, gender, diabetes, hypertension, hypercholesterolemia, congestive heart failure, history of atrial fibrillation, coronary artery disease, family history of coronary artery disease, creatinine levels, chronic obstructive pulmonary disease, peripheral vascular disease, cerebrovascular disease, Ambler score, previous percutaneous coronary intervention, previous valve replacement or repair, previous intrapericardial or vessel	good

								procedure, repeat sternotomy, concomitant atrial fibrillation ablation surgery, concomitant coronary artery bypass grafting, isolated mitral valve surgery, isolated aortic valve surgery, tricuspid valve surgery, multivalve (aortic and mitral) surgery, left ventricular ejection fraction, elective status, New York Heart Association class III or IV, and perioperative medications	
65	CVD	Wang et al. 2015	159	1286	atrial fibrillation	Asia	2.1	congestive heart failure, hypertension, diabetes mellitus, prior stroke/TIA, peripheral vascular disease, previous TE other than stroke/TIA, age ≥75 years, smoking, paroxysmal AF, renal dysfunction, anticoagulation therapy, and sex category	good
66	CVD	Wang et al. 2017	131	10723	patients after PCI	Asia	2	age, gender, diabetes, hypertension, current smoking, PVD, dyslipidemia, CVD, previous PCI, previous CABG, current smoking, hemoglobin before PCI, Ccr<mL/min/1.73 m2, LVEF<40%, syntax score, chroni c total occlusion lesion, B2 or C type lesion, left main involved, transfemoral, and TIMI 2 or 3 flow after PC	good
67	CVD	Wang et al. 2019	862	10942	atrial fibrillation	Asia	2.5	age, heart failure, coronary artery disease, stroke/transient ischemic attack/peripheral thromboembolism, hypertension, diabetes mellitus, hyperlipidemia, history of bleeding, chronic kidney disease: eGFR<60 mL/min/1.73 m2, chronic obstructive pulmonary disease, the usage of warfarin, angiotensin-converting enzyme inhibitor/angiotensin receptor blocker, antiarrhythmic drugs and statin	good
68	CVD	Won et al. 2023	82	5112	PCI	Asia	2	NA	fair
69	CVD	Wu et al. 2009	143	446	heart failure due to acute coronary syndrome	North America	9.4	age, male gender, diabetes, blocker prescribed at hospital discharge, angio- tensin-converting enzyme inhibitor or angiotensin receptor blocker prescribed at discharge, initial creatinine, and hemoglobin	good
70	CVD	Xu et al. 2015	34354	388724	hypertension	Europe	8	age, sex, pre existing medical conditions, smoking, alcohol use, and socioeconomic status, interaction terms between BMI category and preexisting cardiovascular diagnoses, COPD, smoking, and alcohol history. Hazard ratios represent hazards for patients who had no history of smoking, heavy alcohol use, cardiovascular disease, or COPD	good
71	CVD	Yang et al. 2016	622	20694	hypertension	Asia	4.5	sex, age, center, baseline and on-treatment BP, smoking status, alcohol drinking, education, stress, fasting blood glucose, total cholesterol, triglycerides, serum creatinine, homocysteine, albumin	good
72	CVD	Zittermann et al. 2014	880	9133	cardiac surgery	Europe	2	age, sex, smoking status, left ventricular ejection fraction, eGFR, EuroSCORE, type of surgery, on-pump surgery, operative priority, concomitant diagnoses, biochemical parameteres, medications	good
73	Cancer	Arem et al. 2013	299	1400	endometrial cancer	Nort America	10	categorical variables tumor grade, tumor stage, surgery, chemotherapy, race, family history of breast cancer, diabetes, and smoking status	good
74	Cancer	Arem et al. 2017	91	467	endometrial cancer	North America	10.2	age at diagnosis, tumor stage, tumor grade, diabetes, age at menarche, self-reported health status	good
75	Cancer	Baade et al. 2011	458	1779	colorectal cancer	Australia	4.9	age, stage, smoking status, site, treatment, sex, lifestyle and clinical variables, physical activity	good

76	Cancer	Doria-Rose et al. 2006	271	633	colorectal cancer in postmenopausal women	North America	9.4	stratified on age and stage of the disease, and adjusted for postmenopausal hormone use and smoking	good
77	Cancer	Kamineni et al. 2012	97	464	early-stage breast cancer	North America	10	age, stage, and tamoxifen use	good
78	Cancer	Kawai et al. 2016	1418	20090	breast cancer	Asia	6.7	age, living place, detection method, family history of breast cancer, tumor stage, radiation therapy, chemotherapy, endocrine therapy, tumor subtypes, menopausal status, and registered year	good
79	Cancer	Lee et al. 2018	1279	7765	gastric cancer	Asia	6.92	age, sex, TNM stage, smoking, family history of gastric cancer, operation, histology, location, and ASA score	good
80	Cancer	Du Y et al. 2023	1018	1888	nasopharyngeal carcinoma	Asia	5.1	age, sex, smoking and alcohol history, cancer stage, and treatment information	good
81	Cancer	Reichle et al. 2015	4571	8673	cancer	Europe	11.9	age at cancer diagnosis (in years), smoking status, and sex and statistically controlled according to tumor stage and cancer entity (	good
82	Cancer	Troeschel et al. 2020	3855	8330	prostate cancer	North America	7.3	age, treatment, tumor extent, Gleason score, postdiagnosis physical activity, education, diagnosis year	good
83	Cancer	Zhou et al. 2014	346	600	ovarian cancer	North America	10.9	age at baseline, time from study enrollment to ovarian cancer diagnosis, cancer disease stage, histology, hormone therapy use status, smoking status, physical activity, history of diabetes and status in the calcium and vitamin D trial, dietary modification trial, hormone therapy trials, and/or observational study	good
84	Other	Agarval et al. 2011	119	368	hemodialysis patients	North America	2.7	sex, smoking, urea reduction ratio, aspirin use, diabetes mellitus, and serum albumin, number of antihypertensive medications, inferior vena cava diameter in expiration	good
85	Other	Ahmed et al. 2022	569	4834	NAFLD	North America	20	sex, diabetes, HTN, and dyslipidemia	good
86	Other	Allen et al. 2010	4959	11384	after lung transplantation	North America	3.1	BMI strata, age 60, gender, donor-recipient gender mismatch, transplant year, creatinine, history of diabetes, diagnosis of idiopathic pulmonary fibrosis, diagnosis of cystic fibrosis, intensive are unit care before LTx, hospitalization before LTx, bilateral LTx, ischemic time, private insurance/self-pay, and 6-minute walk distance	good
87	Other	Chen et al. 2023	856	11900	schizophrenia	Europe	2.6	age, gender, marital status, ethnicity, employment status, IMD score, smoking status, HoNOS problems, received antipsychotic, antidepressant, manic, antihypertensives, lipid-lowering and antidiabetic medications, and comorbidities of diabetes, hypertension and hyperlipidaemia.	good
88	Other	Dowsey et al. 2018 a	3698	55680	total hip arthroplasty	North America	4	age, gender, race, year of operation, gender, diabetes, primary diagnoses, and ASA category, bilateral surgery, diabetes	good
89	Other	Dowsey et al. 2018 b	6707	106874	total knee arthroplasty	North America	4	age, gender, race, year of operation, gender, diabetes, primary diagnoses, and ASA category, bilateral surgery, diabetes	good
90	Other	Dowsey et al. 2018 c	328	2950	total hip arthroplasty	Australia	5	age, Australian-born, year of operation, gender, diabetes, primary diagnoses, and ASA category, indication for surgery, diabetes	good

91	Other	Dowsey et al. 2018 d	395	3435	total knee arthroplasty	Australia	5	age, Australian-born, year of operation, gender, diabetes, primary diagnoses, and ASA category, indication for surgery, diabetes	good
92	Other	Izumida et al. 2019	1101	4498	metabolically unhealthy	Asia	18.4	sex, age, total cholesterol, smoking status (never, ex-, or current smoker), drinking status, education attainment (<18 years or ≥18 years), married status (yes or no), physical activity index (PAI), and sleeping hours	good
93	Other	Kim et al. 2020	4975	18699	hemodialysis patients	Asia	4	age, sex, smoking, alcohol drinking, regular exercise, low income, previous history of diabetes, hypertension, dyslipidemia, cardiovascular disease, cancer, and BMI or WC	good
94	Other	Lee et al. 2023	212	238	pulmonary fibrosis	North America	2	NA	good
95	Other	Prieto-Alhambra et al. 2014 a	830	4647	hip fracture	Europe	1.2	age, gender, smoking, alcohol intake, oral glucocorticoid use, and Charlson comorbidity index.	good
96	Other	Prieto-Alhambra et al. 2014 b	170	2847	proximal humerus fracture	Europe	1.3	age, gender, smoking, alcohol intake, oral glucocorticoid use, and Charlson comorbidity index.	good
97	Other	Prieto-Alhambra et al. 2014 c	823	21765	osteoporotic nonhip fracture	Europe	1.4	age, gender, smoking, alcohol intake, oral glucocorticoid use, and Charlson comorbidity index.	good

The list of included studies.

1. Baldinger B, Schwarz C, Jaggy C. Cardiovascular risk factors, BMI and mortality in a cohort of Swiss males (1976-2001) with high-sum-assured life insurance cover. J Insur Med. 2006; 38: 44-53.
2. Cohen SS, Park Y, Signorello LB, et al. A pooled analysis of body mass index and mortality among African Americans. PLoS One. 2014; 9: 111980.
3. Freedman DM, Ron E, Ballard-Barbash R, et al. Body mass index and all-cause mortality in a nationwide US cohort. Int J Obes (Lond). 2006; 30: 822-829.
4. Gelber RP, Kurth T, Manson JE, et al. Body mass index and mortality in men: evaluating the shape of the association. Int J Obes (Lond). 2007; 31:1240-1247.
5. Hjartåker A, Adami HO, Lund E, Weiderpass E. Body mass index and mortality in a prospectively studied cohort of Scandinavian women: the women's lifestyle and health cohort study. Eur J Epidemiol. 2005; 20: 747-754.
6. Hu F, Cheng J, Yu Y, et al. Association Between Body Mass Index and All-Cause Mortality in a Prospective Cohort of Southern Chinese Adults Without Morbid Obesity. Front Physiol. 2022; 13: 857787.
7. Katzmarzyk PT, Reeder BA, Elliott S, et al. Body mass index and risk of cardiovascular disease, cancer and all-cause mortality. Can J Public Health. 2012; 103: 147-151.
8. Kee CC, Sumarni MG, Lim KH, et al. Association of BMI with risk of CVD mortality and all-cause mortality. Public Health Nutr. 2017; 20: 1226-1234.
9. Klenk J, Nagel G, Ulmer H, et al. Body mass index and mortality: results of a cohort of 184,697 adults in Austria. Eur J Epidemiol. 2009; 24: 83-91.

10. Lin WY, Tsai SL, Albu JB, et al. Body mass index and all-cause mortality in a large Chinese cohort. CMAJ. 2011; 183: 329-336
11. Manne-Goehler J, Baisley K, Vandormael A, et al. BMI and All-Cause Mortality in a Population-Based Cohort in Rural South Africa. Obesity (Silver Spring). 2020; 28: 2414-2423.
12. Nagai M, Kuriyama S, Kakizaki M, et al. Effect of age on the association between body mass index and all-cause mortality: the Ohsaki cohort study. J Epidemiol. 2010; 20: 398-407.
13. Shimazu T, Kuriyama S, Ohmori-Matsuda K, et al. Increase in body mass index category since age 20 years and all-cause mortality: a prospective cohort study (the Ohsaki Study). Int J Obes (Lond). 2009; 33: 490-496.
14. Song YM, Ha M, Sung J. Body mass index and mortality in middle-aged Korean women. Ann Epidemiol. 2007; 17: 556-563.
15. Thorogood M, Appleby PN, Key TJ, Mann J. Relation between body mass index and mortality in an unusually slim cohort. J Epidemiol Community Health. 2003; 57: 130-133.
16. Zahir SF, Griffin A, Veerman JL, et al. Exploring the association between BMI and mortality in Australian women and men with and without diabetes: the AusDiab study. Diabetologia. 2019; 62: 754-758.
17. Atlantis E, Browning C, Kendig H. Body mass index and unintentional weight change associated with all-cause mortality in older Australians: the Melbourne Longitudinal Studies on Healthy Ageing (MELSHA). Age Ageing. 2010; 39: 643-646.
18. Corrada MM, Kawas CH, Mozaffar F, Paganini-Hill A. Association of body mass index and weight change with all-cause mortality in the elderly. Am J Epidemiol. 2006; 163: 938-949.
19. Li ZB, Wang ML, Dong SY, Sun BR, et al. Effects of body mass index and weight change on mortality in older men with impaired glucose regulation. Exp Gerontol. 2017; 89: 87-92.

20. Takata Y, Ansai T, Soh I, et al. Association between body mass index and mortality in an 80-year-old population. J Am Geriatr Soc. 2007; 55: 913-917.

21. Tamakoshi A, Yatsuya H, Lin Y, et al. BMI and all-cause mortality among Japanese older adults: findings from the Japan collaborative cohort study. Obesity (Silver Spring). 2010; 18: 362-369.

22. Wang YF, Tang Z, Guo J, Tao LX, Liu L, Li HB, Li DT, Guo XH, Yang XH. BMI and BMI Changes to All-cause Mortality among the Elderly in Beijing: a 20-year Cohort Study. Biomed Environ Sci. 2017; 30: 79-87.

23. Wang J, Taylor AW, Zhang T, et al. Association between Body Mass Index and All-Cause Mortality among Oldest Old Chinese. J Nutr Health Aging. 2018; 22: 262-268.

24. Costanzo P, Cleland JG, Pellicori P, et al. The obesity paradox in type 2 diabetes mellitus: relationship of body mass index to prognosis: a cohort study. Ann Intern Med. 2015; 162: 610-618.

25. Edqvist J, Rawshani A, Adiels M, et al. BMI and Mortality in Patients With New-Onset Type 2 Diabetes: A Comparison With Age- and Sex-Matched Control Subjects From the General Population. Diabetes Care. 2018; 41: 485-493.

26. German CA, Laughey B, Bertoni AG, Yeboah J. Associations between BMI, waist circumference, central obesity and outcomes in type II diabetes mellitus: The ACCORD Trial. J Diabetes Complications. 2020; 34: 107499.

27. Hong JS, Kang HC. Body mass index and all-cause mortality in patients with newly diagnosed type 2 diabetes mellitus in South Korea: a retrospective cohort study. BMJ Open. 2022; 12: 048784.

28. Huang HL, Kung CY, Wang SM, et al. Effects of Body Mass Index and Pay-for-Performance Program on Risk of Death in Patients with Type 2 Diabetes: A Nationwide Cohort Study. Int J Environ Res Public Health. 2021; 18: 4648.

29. Lee DH, Ha KH, Kim HC, Kim DJ. Association of Body Mass Index with Risk of Major Adverse Cardiovascular Events and Mortality in People with Diabetes. J Obes Metab Syndr. 2018; 27: 61-70.
30. Lee YB, Kim B, Park J, et al. Early mortality and cardiovascular disease, varied association with body mass index and its changes in insulin-treated diabetes: a nationwide study. Int J Obes (Lond). 2021; 45: 2482-2489.
31. Lin CC, Li CI, Liu CS, et al. Obesity paradox in associations between body mass index and diabetes-related hospitalization and mortality in patients with type 2 diabetes: Retrospective cohort studies. Diabetes Metab. 2019; 45: 564-572.
32. Liu H, Wu S, Li Y, et al. Body mass index and mortality in patients with type 2 diabetes mellitus: A prospective cohort study of 11,449 participants. J Diabetes Complications. 2017; 31: 328-333.
33. Ma J, Wang X, Zheng M, et al. A Multicenter Large-Scale Retrospective Analysis of the Correlation between Body Mass Index and All-Cause Mortality in Patients with Type 2 diabetes Mellitus: A Seven-Year Real-World Study. Endocr Res. 2019; 44: 103-109.
34. Orsi E, Solini A, Penno G, et al. Renal Insufficiency And Cardiovascular Events (RIACE) Study Group. Body mass index versus surrogate measures of central adiposity as independent predictors of mortality in type 2 diabetes. Cardiovasc Diabetol. 2022; 21: 266.
35. Salehidoost R, Mansouri A, Amini M, et al. Body mass index and the all-cause mortality rate in patients with type 2 diabetes mellitus. Acta Diabetol. 2018; 55: 569-577.
36. Vestberg D, Rosengren A, Eeg-Olofsson K, et al. Body mass index as a risk factor for coronary events and mortality in patients with type 1 diabetes. Open Heart. 2018; 5: 000727.
37. Aiumtrakul N, Kittithaworn A, Supasyndh O, et al. Association of body mass index with kidney function and mortality in high cardiovascular risk population: A nationwide prospective cohort study. Nephrology (Carlton). 2022; 27: 25-34.

38. Cai C, Hua W, Ding LG, et al. Association of body mass index with cardiac reverse remodeling and long-term outcome in advanced heart failure patients with cardiac resynchronization therapy. Circ J. 2014; 78: 2899-2907.

39. Curtis JP, Selter JG, Wang Y, et al. The obesity paradox: body mass index and outcomes in patients with heart failure. Arch Intern Med. 2005; 165: 55-61.

40. Faggioni M, Baber U, Afshar AE, et al. Effects of Body Mass Index on Clinical Outcomes in Female Patients Undergoing Percutaneous Coronary Intervention With Drug-Eluting Stents: Results From a Patient-Level Pooled Analysis of Randomized Controlled Trials. JACC Cardiovasc Interv. 2018; 11: 68-76.

41. Feng X, Zhang C, Jiang L, et al. Body mass index and mortality in patients with severe coronary artery diseases: A cohort study from China. Nutr Metab Cardiovasc Dis. 2021; 31: 448-454.

42. Haass M, Kitzman DW, Anand IS, Miller A, Zile MR, Massie BM, Carson PE. Body mass index and adverse cardiovascular outcomes in heart failure patients with preserved ejection fraction: results from the Irbesartan in Heart Failure with Preserved Ejection Fraction (I-PRESERVE) trial. Circ Heart Fail. 2011; 4: 324-31.

43. Hamaguchi S, Tsuchihashi-Makaya M, Kinugawa S, et al. Body mass index is an independent predictor of long-term outcomes in patients hospitalized with heart failure in Japan. Circ J 2010; 74: 2605-2611.

44. Hwang IC, Choi HM, Yoon YE, et al. Body Mass Index, Muscle Mass, and All-Cause Mortality in Patients With Acute Heart Failure: The Obesity Paradox Revisited. Int J Heart Fail. 2022; 4: 95-109.

45. Inoue H, Kodani E, Atarashi H, et al. Impact of Body Mass Index on the Prognosis of Japanese Patients With Non-Valvular Atrial Fibrillation. Am J Cardiol. 2016; 118: 215-221.

46. Jong CB, Li HY, Pan SL, et al. Relationship Between Body Mass Index, Antidiabetic Agents, and Midterm Mortality in Patients With Both Type 2 Diabetes Mellitus and Acute Coronary Syndrome. J Am Heart Assoc. 2019; 8: 011215
47. Kenchaiah S, Pocock SJ, Wang D, et al. Body mass index and prognosis in patients with chronic heart failure: insights from the Candesartan in Heart failure: Assessment of Reduction in Mortality and morbidity (CHARM) program. Circulation. 2007; 116: 627-636.
48. Masoudkabir F, Yavari N, Jameie M, et al. The association between different body mass index levels and midterm surgical revascularization outcomes. PLoS One. 2022; 17: 0274129.
49. Moholdt T, Lavie CJ, Nauman J. Interaction of Physical Activity and Body Mass Index on Mortality in Coronary Heart Disease: Data from the Nord-Trøndelag Health Study. Am J Med. 2017; 130: 949-957.
50. Nezu M, Ueda S, Uchida K, et al. Association between body mass index and long-term clinical outcomes in patients with non-valvular atrial fibrillation taking oral anticoagulants. Heart Vessels. 2023; 38: 551-561.
51. Olsen TS, Dehlendorff C, Petersen HG, Andersen KK. Body mass index and poststroke mortality. Neuroepidemiology. 2008; 30: 93-100.
52. Shiga T, Kohro T, Yamasaki H, et al. Body Mass Index and Sudden Cardiac Death in Japanese Patients After Acute Myocardial Infarction: Data From the JCAD Study and HIJAMI-II Registry. J Am Heart Assoc. 2018; 7: 008633.
53. Tokarek TA, Dziewierz A, Sorysz D, et al. The obesity paradox in patients undergoing transcatheter aortic valve implantation: is there any effect of body mass index on survival? Kardiol Pol. 2019; 77: 190-197.
54. Vaduganathan M, Lee R, Beckham AJ, et al. Relation of body mass index to late survival after valvular heart surgery. Am J Cardiol. 2012; 110: 1667-1678.

55. Wang HJ, Si QJ, Shan ZL, et al. Effects of body mass index on risks for ischemic stroke, thromboembolism, and mortality in Chinese atrial fibrillation patients: a single-center experience. PLoS One. 2015; 10: 0123516.
56. Wang H, Gao Z, Zhao X, et al. Association of body mass index with mortality in Chinese patients after percutaneous coronary intervention: A large single-center data. Cardiovasc Ther. 2017; 35.
57. Wang L, Du X, Dong JZ, et al. Body mass index and all-cause mortality in patients with atrial fibrillation: insights from the China atrial fibrillation registry study. Clin Res Cardiol. 2019; 108: 1371-1380.
58. Won KB, Shin ES, Kang J, et al. Body Mass Index and Major Adverse Events During Chronic Antiplatelet Monotherapy After Percutaneous Coronary Intervention With Drug-Eluting Stents - Results From the HOST-EXAM Trial. Circ J. 2023; 87: 268-276.
59. Wu AH, Eagle KA, Montgomery DG, et al. Relation of body mass index to mortality after development of heart failure due to acute coronary syndrome. Am J Cardiol. 2009; 103: 1736-1740.
60. Xu W, Shubina M, Goldberg SI, Turchin A. Body mass index and all-cause mortality in patients with hypertension. Obesity (Silver Spring). 2015; 23: 1712-1720.
61. Yang W, Li JP, Zhang Y, et al. Association between Body Mass Index and All-Cause Mortality in Hypertensive Adults: Results from the China Stroke Primary Prevention Trial (CSPPT). Nutrients. 2016; 8: 384.
62. Zittermann A, Becker T, Gummert JF, Börgermann J. Body mass index, cardiac surgery and clinical outcome. A single-center experience with 9125 patients. Nutr Metab Cardiovasc Dis. 2014; 24: 168-175.
63. Arem H, Park Y, Pelser C, et al. Prediagnosis body mass index, physical activity, and mortality in endometrial cancer patients. J Natl Cancer Inst. 2013; 105: 342-349.

64. Arem H, Chlebowski R, Stefanick ML, et al. Body mass index, physical activity, and survival after endometrial cancer diagnosis: results from the Women's Health Initiative. Gynecol Oncol. 2013; 128: 181-186.
65. Baade PD, Meng X, Youl PH, et al. The impact of body mass index and physical activity on mortality among patients with colorectal cancer in Queensland, Australia. Cancer Epidemiol Biomarkers Prev. 2011; 20: 1410-1420.
66. Doria-Rose VP, Newcomb PA, Morimoto LM, et al. Body mass index and the risk of death following the diagnosis of colorectal cancer in postmenopausal women (United States). Cancer Causes Control. 2006; 17: 63-70.
67. Kamineni A, Anderson ML, White E, et al. Body mass index, tumor characteristics, and prognosis following diagnosis of early-stage breast cancer in a mammographically screened population. Cancer Causes Control. 2013; 24: 305-312.
68. Kawai M, Tomotaki A, Miyata H, et al. Body mass index and survival after diagnosis of invasive breast cancer: a study based on the Japanese National Clinical Database-Breast Cancer Registry. Cancer Med. 2016; 5: 1328-1340.
69. Lee JH, Park B, Joo J, et al. Body mass index and mortality in patients with gastric cancer: a large cohort study. Gastric Cancer. 2018; 21: 913-924.
70. Reichle K, Peter RS, Concin H, Nagel G. Associations of pre-diagnostic body mass index with overall and cancer-specific mortality in a large Austrian cohort. Cancer Causes Control. 2015; 26: 1643-1652.
71. Troeschel AN, Hartman TJ, Jacobs EJ, et al. Postdiagnosis Body Mass Index, Weight Change, and Mortality From Prostate Cancer, Cardiovascular Disease, and All Causes Among Survivors of Nonmetastatic Prostate Cancer. J Clin Oncol. 2020; 38: 2018-2027.
72. Zhou Y, Chlebowski R, LaMonte MJ, et al. Body mass index, physical activity, and mortality in women diagnosed with ovarian cancer: results from the Women's Health Initiative. Gynecol Oncol. 2014; 133: 4-10.
73. Agarwal R. Body mass index-mortality paradox in hemodialysis: can it be explained by blood pressure? Hypertension. 2011; 58: 1014-1020.

74. Ahmed OT, Gidener T, Mara KC, et al. Natural History of Nonalcoholic Fatty Liver Disease With Normal Body Mass Index: A Population-Based Study. Clin Gastroenterol Hepatol. 2022; 20: 1374-1381.

75. Allen JG, Arnaoutakis GJ, Weiss ES, Merlo CA, Conte JV, Shah AS. The impact of recipient body mass index on survival after lung transplantation. J Heart Lung Transplant. 2010; 9: 1026-1033.

76. Chen J, Perera G, Shetty H, et al. Body mass index and mortality in patients with schizophrenia spectrum disorders: a cohort study in a South London catchment area. Gen Psychiatr. 2022; 35: 100819.

77. Dowsey MM, Choong PFM, Paxton EW, et al. Body Mass Index Is Associated With All-cause Mortality After THA and TKA. Clin Orthop Relat Res. 2018; 476: 1139-1148.

78. Izumida T, Nakamura Y, Ishikawa S. Impact of body mass index and metabolically unhealthy status on mortality in the Japanese general population: The JMS cohort study. PLoS One. 2019; 14: 0224802.

79. Kim CS, Han KD, Choi HS, et al. Association of Body Mass Index and Waist Circumference with All-Cause Mortality in Hemodialysis Patients. J Clin Med. 2020; 9: 1289.

80. Lee JS, Martin-Schwarze A, Freiheit E, et al. Real-World Clinical Outcomes Based on Body Mass Index and Annualized Weight Change in Patients with Idiopathic Pulmonary Fibrosis. Adv Ther. 2023; 40: 691-704.

81. Prieto-Alhambra D, Premaor MO, Avilés FF, et al. Relationship between mortality and BMI after fracture: a population-based study of men and women aged ≥40 years. J Bone Miner Res. 2014; 29: 1737-1744.

82. Du Y, Feng R, Chang ET, et al. Body mass index and body shape before treatment and nasopharyngeal carcinoma prognosis: A population-based patient cohort study in southern China. Int J Cancer. 2023; 153: 290-301.
